# Wood Bio-Adhesives Made by Polymerizing Oxidized Starch with Deep Eutectic Solvent-Modified Lignin

**DOI:** 10.3390/polym17223023

**Published:** 2025-11-14

**Authors:** Hamed Younesi-Kordkheili, Antonio Pizzi

**Affiliations:** 1 Department of Wood and Paper Sciences, Faculty of Natural Resources, Semnan University, Semnan 35131-19111, Iran; 2 Laboratoire d’Etude et de Recherche sur le Materiau Bois (LERMAB), University of Lorraine, Blvd des Aiguielles, 54000 Nancy, France

**Keywords:** lignin, oxidized starch, DES-modified lignin, urea, wood bio-adhesive, physical and mechanical properties

## Abstract

In the present work, a new bio-sourced adhesive system based on deep eutectic solvent-modified lignin and oxidized starch (OSTL) resin is presented. For this purpose, unmodified and choline chloride–Zinc chloride (ChCl–ZnCl_2_) deep eutectic solvent modified lignin at different contents (10%, 20%, and 30%) were used to prepare the OSTL resin. Ammonium persulfate (APS) was the oxidizer employed for the oxidation of starch, and urea was used as a low cost and effective crosslinker agent in the OSTL resin. FTIR analysis indicated that the content of carboxyl and carbonyl groups changed after the curing of the OSTL resin compared to oxidized starch (OST). DSC analysis indicated that the curing temperature of the OSTL resin containing DES-modified lignin was lower than that for unmodified lignin. Also, greater dimensional stability and mechanical strength could be achieved by increasing the amount of DES-treated lignin in the OSTL wood adhesive from 10 to 30 wt%. Based on the findings of this research, the physical and mechanical properties of the particleboard panels bonded with this type of bio-adhesive were acceptable according to the relevant standards. Additionally, urea can thus be used as a good cross-linker, not only to crosslink just OST, but also to connect DES-modified lignin and oxidized starch molecules. Under the conditions used, particleboards bonded with an oxidized starch–urea–pristine lignin adhesive presented decreasing internal bond (IB) strength with an increasing proportion of lignin. Conversely, when the same adhesive using DES-modified lignin was used, the internal bond (IB) strength improved with the increasing proportion of DES-modified lignin. At 30% proportions of lignin, the oxidized starch–urea–DES-modified lignin presented a 27% improvement in strength. Finally, it can be noted that this work brings a new insight to the development and application of lignin-based bio-adhesives to bond wood-based panels.

## 1. Introduction

Nowadays, formaldehyde-based resins such as urea–formaldehyde resins (UF) and phenol–formaldehyde resins (PF) are the main significant wood panel adhesives used worldwide. Despite all the unique advantages of formaldehyde-based resins, their main defect is the release of formaldehyde from the wood panels bonded with them. Another shortcoming of formaldehyde-based wood adhesives is that their production relies on petroleum resources. For these reasons, nowadays, bio-based wood adhesives such as tannins, soy, chitosan, carbohydrates, and others are being rapidly developed [1,2]. Among all the biomaterials available, lignin shows good potential to be used in wood adhesive preparation [3].

**Figure 1:**
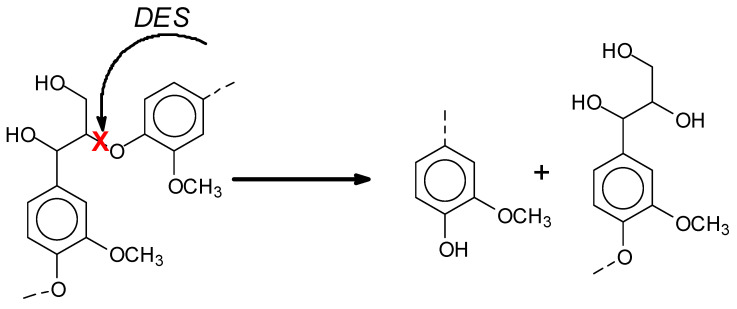
Effect of DES action on lignin by cleavage of the lignin β-O-4 inter-units linkage (red cross).

Lignin is the second largest biomass resource in the world. The total annual production of industrial lignin produced by the pulp and paper industries can reach 6 × 10^10^ tons [4]. The heterogeneity and complicated macromolecular structure, together with low reactivity, makes the direct utilization of lignin in the wood adhesive impossible [5,6]. For this reason, lignin-based wood adhesives face challenges such as low bonding strength, which have significantly limited their applications. Hence, a proper method to improve the properties of pristine lignin for its use as a wood adhesive is necessary. So far, different methods such as hydrolysis, glyoxalation, phenolation, pyrolysis, hydrogenolysis, oxidation and biodegradation have been proposed for lignin modification. Among these, deep eutectic solvents (DESs) are a new method that can provide a mild acid–base catalytic mechanism, causing the cleavage of the ether bonds between lignin phenylpropane units, leading to lignin partial depolymerization as well as partial demethylation, as already proved by other research groups and shown in Figure 1 [7,8].

The cleavage of ether bonds in the lignin structure after DES modification can decrease the lignin average molecular weight and its polydispersity index, along with causing a significant increase in its homogeneity and on the number of phenolic–OH groups and a decrease in the methoxy groups present [9].

Conversely, the combination of lignin with formaldehyde to obtain a wood adhesive is not desirable and cannot solve the formaldehyde emission problems. For this reason, to obtain a green adhesive based on lignin, the use of a nontoxic aldehyde is needed. Therefore, many researchers have explored the potential use of petroleum-based aldehydes such as furfural, glutaraldehyde, and glyoxal to replace formaldehyde [10]. Although the toxicity of these aldehydes is lower than that of formaldehyde, they are nonetheless expensive, causing an increased cost for the adhesive. Hence, finding a new aldehyde at lower cost and with good performance to prepare bio-adhesives based on lignin is of considerable importance.

The use of natural green sugar-based materials such as starch, sucrose, glucose, and cellulose to serve as an excellent feed-stock for sugar-based aldehydes is growing. Among sugar-based materials, starch is an abundant, inexpensive natural polymer [11]. However, similar to lignin, starch also has low reactivity and typically requires chemical or physical modification for optimal application. Among suggested modifications, oxidation is one of the most common chemical treatments. Starch and other carbohydrates that undergo oxidation by different oxidizing agents such as H_2_O_2_, sodium periodate and others have a relatively long but recent history of use in wood adhesives to generate aldehyde groups that are non-toxic and non-volatile on the structural skeletons of polymeric carbohydrates [12,13,14,15,16], and are even able to directly oxidize the surface of wood to generate aldehydes from the cellulose and hemicellulose of the wood surface to improve the performance of the bioadhesive [17]. Compared to strong oxidizing agents which can cause significant structural damage to the sugars, persulfates (PS) are inexpensive and relatively mild oxidizing agents, and they have recently been used for the first time as the most appropriate agent to oxidize carbohydrate adhesives for wood [18]. Previous works have shown that PS can break the glycosidic bonds in the starch’s molecular structures, leading to oxidation of the hydroxyl groups at positions 2 and 3 to aldehydes [19]. Therefore, in this study, ammonium persulfate (APS) was chosen as the oxidizing agent to oxidize the active hydroxyl groups in starch molecules to aldehyde. The aldehyde groups can react with the active hydrogen atoms on the phenolic rings of lignin molecules to form lignin-based adhesives. Previous works have shown that the use of cross-linking modifiers is a helpful method to obtain lignin-oxidized starch adhesives with good physical and mechanical properties. The use of urea to cross-link adhesives made of oxidized starch alone, without any use of formaldehyde, was pioneered by Zhao et al. [20]. For this reason, in the current research, urea, as a low cost, nontoxic and effective material, was used to improve the crosslinks between DES-modified nanolignin and oxidized starch. Hence, the aim of this research was to synthesize a new bio-wood adhesive using oxidized starch and DES-modified lignin, with the presence of urea as a crosslinking agent.

## 2. Materials and Methods

### 2.1. Materials

Bagasse Soda black liquor with pH = 13 and 40% solid content as the source of lignin was prepared by Pars Company (Haft Tepe, Iran). For that purpose, Soda lignin was extracted from black liquor using the sulphuric acid method. The chemical materials used in this research, such as urea, ammonium persulfate (APS) and soluble starch, were purchased from the Sigma Aldrich chemical company. In this research, bio-aldehydes, as soluble amylose-rich types of starch, were used to prepare lignin-based adhesives.

### 2.2. Methods

#### 2.2.1. Oxidation of Starch

Starch was oxidized according to a method reported by Liu et al. (2025) [21]. At a temperature of 60 °C, a certain mass ratio of starch (ST) was added to a three-necked flask equipped with a mechanical stirrer to prepare a 50 wt% aqueous solution of starch. After the starch was fully and evenly homogenized, a certain amount of ammonium persulfate ((NH_4_)_2_S_2_O_8_) as the oxidizing agent was charged and stirred for a certain period of time. After the reaction was complete, a filtered portion was exposed to freeze-drying to obtain the oxidized starch (OST).

#### 2.2.2. Modification of Lignin

Lignin was modified according to a method reported by Hong et al. (2016) [22]. Dried choline chloride and zinc chloride were mixed in an Erlenmeyer flask (the molar ratio of choline chloride to zinc chloride was 1:2, ChCl–ZnCl_2_). Lignin and the prepared ZnCl_2_–choline chloride DES (mass ratio lignin:DES was 1:10) were mixed in a water bath under continuous magnetic stirring for 2 h at 90 °C, until the mixture became transparent and homogeneous. After complete dissolution, the lignin was precipitated and regenerated through the addition of water. The regenerated lignin (DL sample) was separated by centrifugation and washed with distilled water until the conductivity was close to that of distilled water. The DL samples were collected and dried at 45 °C. Then, the DL samples were dried under vacuum in an oven at 50 °C for 24 h. The yield of regenerated lignin was about 66% by weight.

#### 2.2.3. Preparation of Oxidized Starch–Lignin (OSTL) Wood Adhesives

To prepare the OSTL adhesive, 10%, 20% and 30% unmodified/modified lignin (based on the dry starch mass) was added to the prepared OST solution (50%) and stirred for 60 min. In the next step, 10 wt% urea (based on the starch mass) as a crosslinking agent was added to the mixture by vigorously stirring with a magnet rotor for 90 min at room temperature. Then, the temperature of the water bath was raised to 90 °C and the reaction was continued for 100 min. Finally, the reaction mixture was then cooled to room temperature to obtain the OSTL adhesive.

#### 2.2.4. Physicochemical Properties of the Synthesized Resin

Physicochemical properties (pH, viscosity and Specific Gravity (SPG)) of the prepared resins were measured according to related standard methods. The percentage of non-volatile content (solid content) of the adhesives was determined as per the China National Standard GB/T 14074-2017 [23]. The specific gravity of the resin solution was obtained using a hydrometer, and the viscosity was also measured using a hydrometer.

#### 2.2.5. Fourier Transform Infrared Spectrometry (FTIR)

The changes in the chemical structure of starch after oxidation and the structure of OST/DES-modified lignin-cured resin were analyzed by Fourier Transform Infrared spectrometry (FTIR) (Shimadzu, FTIR 8400S, Nakagyo, Japan). FTIR spectra were obtained from KBr pellets with 1 wt% of the powdered resin at wave numbers in the 400 and 4000 cm^−1^ range. The obtained spectra were normalized at a constant peak.

#### 2.2.6. Matrix-Assisted Laser Desorption Ionization Time-of-Flight Mass Spectrometry (MALDI ToF)

The samples for MALDI Tof mass spectrometry analysis were prepared by reacting the same proportions of DES-modified lignin with urea, as in Section 2.2.4 above, under exactly the same conditions but in the absence of starch. The resulting product was dissolved in 1 mL of a 50:50 *v*/*v* acetone/water solution. Then, 10 mg of this solution was added to 10 μL of a 2,5–dihydroxy benzoic acid (DHB) matrix. The samples allotted locations on the analysis plaque of the spectrometer were first covered with 2 μL of a 0.1 M NaCl solution in 2:1 *v*/*v* methanol/water. They were then dried. Afterwards, on the exact place on the metal holder plaque of the spectrometer, 1 μL of sample solution was added, followed by drying again. The MALDI-TOF spectra were obtained using an Axima-Performance mass spectrometer from Shimadzu Biotech (Kratos Analytical, Shimadzu Europe Ltd., Manchester, UK). The tuning mode used was a linear polarity-positive one. Overall, 1000 profiles per sample with 2 shots accumulated per profile were used for the spectral analysis measurements. A range of ±1 Da was the precision of the spectrum.

#### 2.2.7. Differential Scanning Calorimetry (DSC) Analysis

The changes in curing temperature of the adhesive containing DES-modified lignin compared to the resin with unmodified lignin were determined by a NETZSCH DSC 200 F3Model thermal analyzer. To determine the curing temperature of the resins, about 5 mg of freeze-dried sample was added to the aluminum pan. The samples were then heated from ambient temperature (25 °C) to 190 °C under a nitrogen atmosphere. The DSC scans were recorded at a heating rate of 10 °C/min under a nitrogen atmosphere with a flow rate of 60 mL/min.

#### 2.2.8. Particleboard Manufacturing

The manufacturing of the particleboards was performed according to the method of Younesi-Kordkheili et al. (2017) [24]. A forming mold frame (35 × 35 × 1 cm) was placed on a stainless steel caul plate to control thickness. The surfaces of each plate were sprayed with a mineral oil releasing agent to ease demolding of the panel after hot pressing. The type of wood used in particleboard production was Beech (*Fagus orientalis*). Dried particles were then blended with the prepared resins in a rotating drum-type mixer fitted with a pneumatic spray gun. Then, compounded material was poured into the frame and spread to fill the frame evenly. When forming was complete, the top caul plate was placed on the top of the mat, and the entire assembly was placed into an oil-heated press that was used for compression molding. The temperature of the press plates was maintained at 200 °C at a maximum pressure of 25 bar pressure. The actual set pressure applied by the hot press to make the board was 25 bars. The pressure and temperature were completely controlled during the pressing time. Pressing of the compound material was carried out, respectively, in two stages, with hot and cold presses for 7 min. One-layer particleboards were manufactured in this research. A 5 cm wide edge of each board was trimmed to remove the low-density and poorly bonding areas of the boards. Three panels were manufactured for each formulation. The nominal thickness and density of manufactured panels was 16 mm and 750 kg/m^3^, respectively. Preparation of the samples for mechanical tests and water absorption measurements started 24 h after pressing.

#### 2.2.9. Panel Testing

All physical and mechanical tests of the particleboards prepared were carried out to the appropriate standard methods. The tests performed on the specimens were internal bond strength (IB-tensile strength perpendicular to the panel plane) and static bending (flexural modulus and flexural strength) according to EN 312 [25]. Short-term (24 h) water absorption and thickness swelling was performed according to ASTM 4442 [26]. The samples were conditioned at a temperature of (23 ± 2) °C and a relative humidity of (60 ± 5) % for two weeks. Five specimens were tested for each panel.

#### 2.2.10. Statistical Analysis

The effects of the unmodified and modified lignin content on the panels’ properties were evaluated by two-way analysis of variance (two-way ANOVA) at a 95% confidence level using SPSS software version 21.

## 3. Results and Discussion

### 3.1. FTIR Analysis

**Figure 2:**
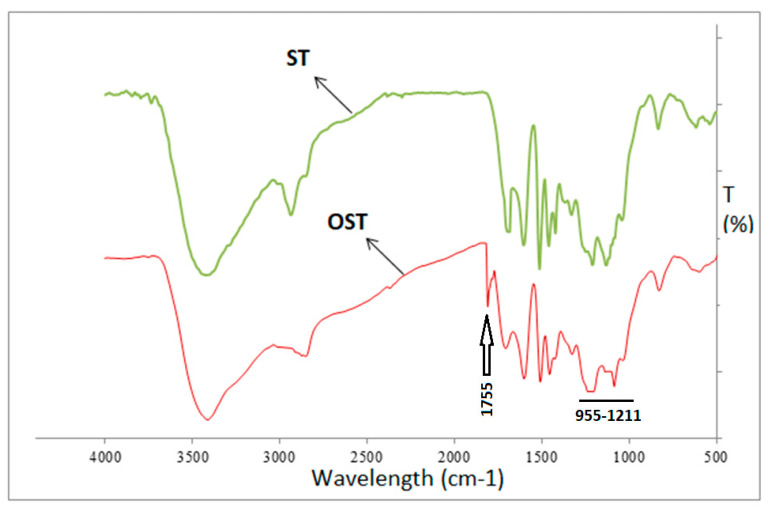
FTIR analysis of the starch (ST) and oxidized starch (OST).

**Figure 3:**
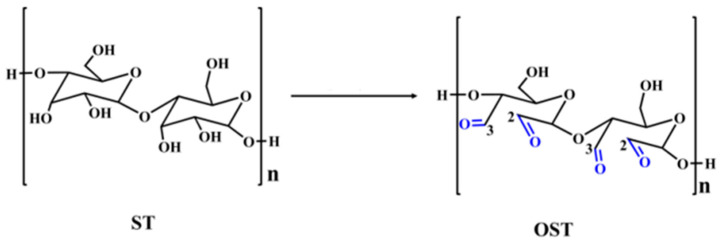
The schematic of the oxidation reaction of starch.

The infrared spectra of the starch (ST) and oxidized starch (OST) are shown in Figure 2. Although a quantitative comparison in FTIR spectra is very difficult and the accuracy of this comparison is low, the lower peaks at 3420–3440 cm^−1^ in the oxidized starch might indicate that compared to unmodified starch, oxidized starch has fewer hydroxyl groups. The comparison of the spectra shows that the FTIR spectrum of OST presents a new peak at 1755 cm^−1^, which is attributed to the stretching vibration of carbonyl groups (C=O) resulting from the oxidation. Thus, this confirms the formation of aldehyde groups after oxidation. Additionally, Figure 2 shows that the intensity of transmittance between the 955 and 1211 cm^−1^ bands in OST decreased compared to ST. This indicates that the molecular chain fragmentation and depolymerization of starch molecules during the oxidation process has probably occurred. The schematic of the oxidation reaction of starch is shown in Figure 3.

It must be noted that not only does starch oxidation generate aldehyde groups, as shown in Figure 3 [20,27,28], but the hydroxyl group at carbon 6 of starch (-CH_2_OH) can also be partially oxidized to an aldehyde (-CHO) group, further increasing the possibility of reaction with other materials [28].

**Figure 4:**
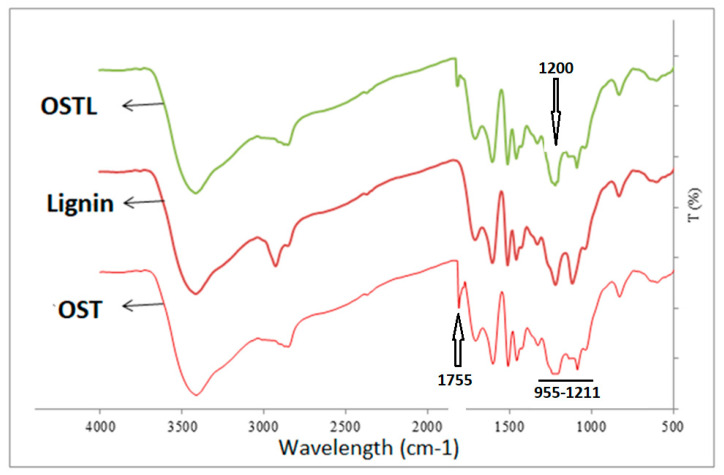
The FTIR spectra of oxidized starch (OST), lignin (L), and synthesized OSTL resin.

**Figure 5:**
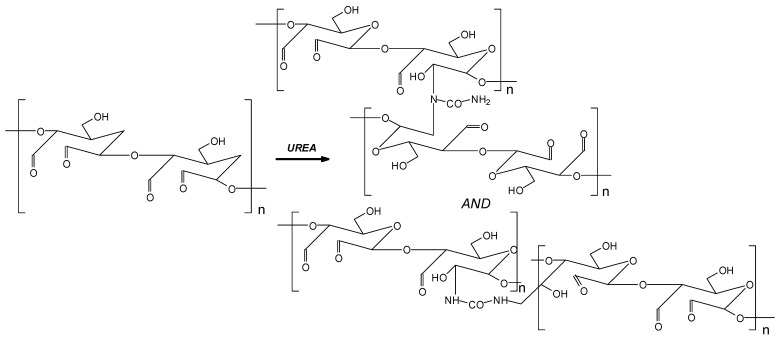
The function of urea as a cross-linker of oxidized starch [16].

Conversely, the FTIR spectra of oxidized starch (OST), lignin (L), and synthesized OSTL resin, after curing, are displayed in Figure 4. This Figure indicates that the peak at 1755 cm^−1^, which is attributed to carbonyl (C=O) groups in OSTL resin, is lower than OST. This reduction probably can be attributed to the consumption of aldehyde groups in reactions during the curing process. The reaction of oxidized starch with urea can be represented as shown in the reaction scheme in Figure 5; namely, the urea functions as a cross-linker of the oxidized starch by reacting with the aldehyde groups generated from the oxidation of vicinal C-OH groups with the consequent cleaving of the C-C bond of the COH-COH covalent coupling.

**Figure 6:**
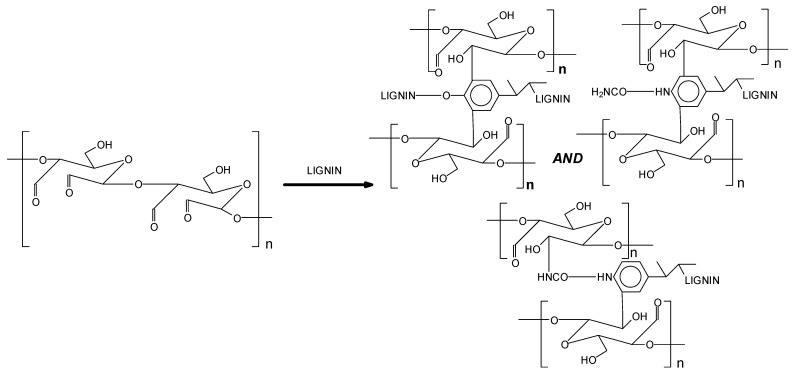
Schematic showing the range of the cross-linking reactions capable of occurring among oxidized starch, urea and DES-modified lignin.

Figure 4 also indicates that the content of carboxyl groups at the 1200 cm^−1^ band increased in terms of OSTL resin rather OST. This signifies that the present carboxyl groups can undergo esterification reactions with hydroxyl groups in lignin during curing. Therefore, the reaction between oxidized starch and DES-modified lignin involves a reaction between aldehyde groups and active sites in the modified lignin. These processes can probably increase the degree of crosslinking in the OSTL adhesive, thereby enhancing the bond strength of the adhesive. Thus, the reaction of the oxidized starch reacted with urea can link with the lower-molecular-weight fragments of the DES-treated lignin, both by reacting some of the aldehydes groups generated on the oxidized starch directly with sites on some of the aromatic rings of lignin units, and also through the reaction of the urea linked to the starch on the phenolic –OHs generated by the DES treatment when links between units are cleaved by this treatment. A simple scheme illustrating these reactions is shown in Figure 6.

Figure 6 shows that the types of links formed are of three main types: (i) just the starch cross-linked by urea [20], (ii) the starch-generated aldehydes due to the oxidation reaction on available reactive aromatic sites of the lignin, as on all phenolic reactive species sites [29], and (iii) the reaction of the –NH_2_ groups of urea substituting some of the phenolic –OHs of lignin and then reacting with the oxidized starch-generated aldehydes. This last reaction is well known for amine groups [30], and does also occur but to a much lesser extent also for amide groups. Thus, urea is an active monomer copolymerizing with the other two materials used to increase the level of cross-linking of the adhesive system. The last cross-link type indicated above contributes less than the other two to the total density of cross-linking of the adhesive system.

**Figure 7:**
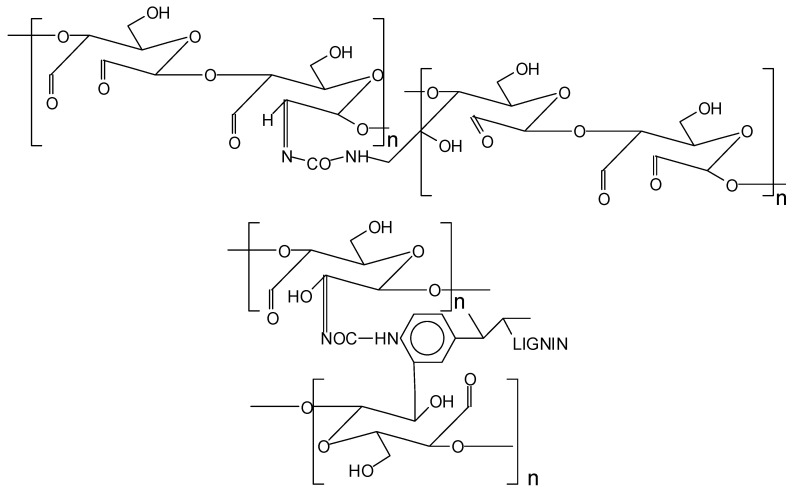
Example of structures likely to occur if imino linkages are present in cross-linked oxidized starch and cross-linked oxidized starch–urea–DES-modified lignin.

It must be clearly pointed out that previous research has indicated that not only does aminal (N-C-N) cross-linking occur, but imine (C=N) cross-linking also occurs between starch chains [17], and as a consequence this might also be likely to occur between starch chains and urea-linked lignin. While this was demonstrated for the reaction of amines with oxidized starch [17], the same reaction, while possible, might be less easy to bring about in the case of an amide such as urea rather than with an amine, simply because in the case of an amide, catalysts are generally needed to obtain an imine [31]. It must equally be pointed out that part of the starch -CH_2_OH of the glucose C6 groups on the starch chain has also been shown to be oxidized to aldehyde, but in this case only amino groups and not imine groups were found to appear to occur between urea and oxidized starch on this starch site [28]. To illustrate the possibility of imino groups involved in the cross-linked structure of starch just oxidized with urea, as well as in the cross-linked oxidized starch–urea–DES-modified lignin system, the types of likely structures are shown in Figure 7.

**Figure 8:**
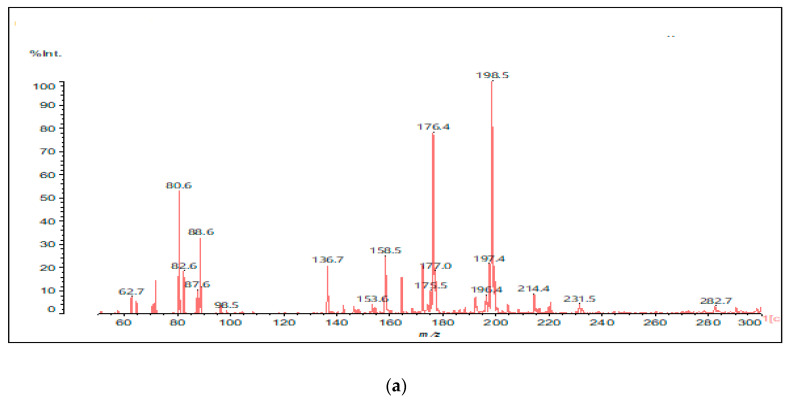

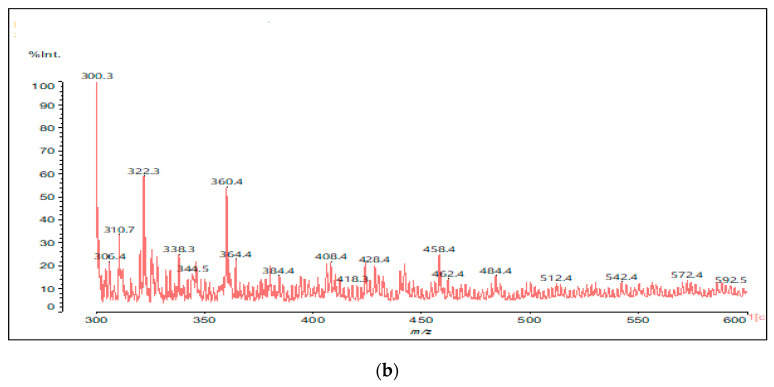
MALDI ToF spectra of DES-modified lignin reacted with urea. (**a**) The 60 Da to 300 Da range. (**b**) The 300 Da to 600 Da range.

To confirm that the reaction of the –NH_2_ groups of urea substituting some of the phenolic –OHs of lignin, although minor, does occur, a MALDI ToF analysis of urea reacted with DES-modified lignin was carried out. Figure 8a,b show the relevant MALDI ToF spectra.

**Figure 9:**
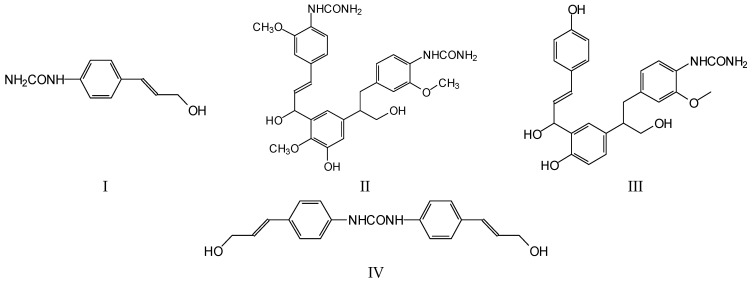
Examples of chemical species obtained by reaction of urea with DES-modified lignin as determined by MALDI ToF spectrometry.

Figure 8 and Figure 9 show, together with the unreacted lignin species, some lignin species reacted with urea through the substitution of one or even two phenolic –OH groups, namely the peaks at 214 Da, 322 Da, 345 Da, 408 Da, 458–462 Da, 484 Da, 502 Da, 542 Da, 572 Da and 592 Da. The table of the total species identified is presented in Table S1 in the Supplementary Material. Some of the species reacted with one or even two urea molecules, such as the occurrence of species such I, II and III, which are shown as examples, and lignin units were even linked by a urea bridge, such as in structure IV (Figure 9).

### 3.2. DSC Analysis

**Figure 10:**
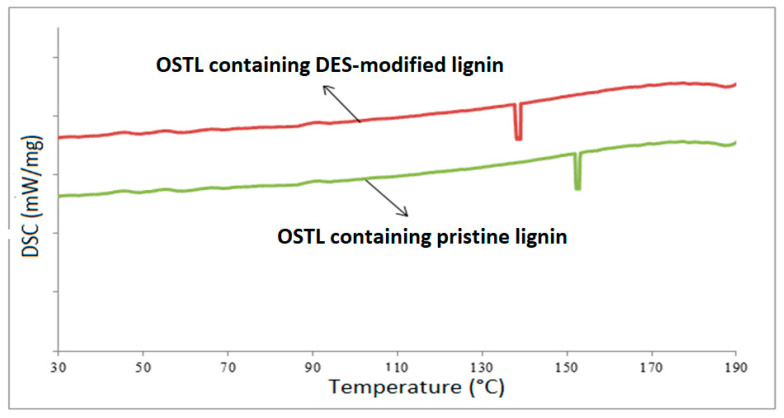
DSC curve of the OSTL resins containing 20% pristine and DES-modified lignin.

The thermal behavior of synthesized OSTL resins containing 20% pristine and modified lignin was performed using DSC analysis (Figure 10). The OSTL resin containing pristine lignin exhibited a distinct exothermic peak about 157 °C. However, the adhesive sample with DES-modified lignin showed its peak at 138 °C. It can be seen that the temperatures of the obtained exothermic peaks during curing of the adhesive vary depending on the type of lignin used. This clearly infers that the treatment of lignin by DES plays an important role in influencing the curing reaction of the adhesive. For the DES treatment of lignin, a lower heat is required for curing. The good performance of DES-modified lignin is probably attributed to its abundant phenolic hydroxyl groups on the molecular chains of the lignin molecules generated by the decrease in the lignin molecular weight by cleavage of the β-O-4 linkages between lignin units. Previous studies indicated that the aldehyde groups can perform additional reactions with the active sites on lignin, such as phenolic reactive sites [21]. Furthermore, it can be noted that from an economic perspective, the modification of lignin with DES before its use in the preparation of a lignin–starch bio-adhesive can produce a resin with suitable curing temperature.

### 3.3. Mechanical and Physical Properties of the Particleboards Prepared

Table 1 shows the mechanical properties of the particleboard panels prepared in this research work. It can be seen that increasing the proportion of unmodified lignin from 10% to 30% progressively decreases the IB strength and flexural properties of the particleboard panels prepared. However, conversely, different results can be observed for the particleboard panels made using DES-modified lignin. The resins with 30% DES-modified lignin had the highest internal bond (IB) strength (0.44 MPa) and bending strength (12.2 MPa), while the panels containing 30 wt% unmodified lignin had the weakest IB strength (0.27 MPa) and bending strength (5.5 MPa) among all the panels prepared. Younesi-Kordkheili et al. (2024) have already shown that the addition of DES-modified lignin to phenol-formaldehyde (PF) resins and bio-adhesives based on lignin increases the mechanical strength of the panels [29].

Conversely, Table 1 shows that the particleboard panels prepared from OSTL resin with DES-modified lignin have a higher IB and bending strength compared to those made with unmodified lignin. The panels with 10, 20 and 30 wt% OSTL containing DES-modified lignin had 11, 32 and 62% higher IB strength and 20, 65 and 120% greater bending strength than the panels with OSTL containing unmodified lignin, respectively. This can be attributed to the differences in physicochemical properties of the OSTL resins with the DES-modified lignin compared to those with pristine lignin (Table 1). Moreover, the improvement in the chemical reactivity of the lignin due to the application of the DES modification can increase the level of cross-linking between the components of the OSTL resin, resulting not only in an increase in the cohesive strength of the resin, but also an improvement in the mechanical strength of the associated particleboard panels.

The bending modulus of the panels prepared is also reported in Table 1. The particleboard panels containing OSTL with DES-modified lignin had 13, 54 and 84 wt% greater bending modulus than the panels with unmodified lignin, respectively. The higher bending modulus of the panels made from OSTL resin with DES-modified lignin can be related to the higher chemical reactivity of the DES-modified lignin. As DES is able to increase the phenolic hydroxyl content by selectively depolymerizing lignin through the ether (β-O-4) link, cleavage can help to improve the bending modulus of the panels prepared [32,33]. The intrinsic bending modulus of lignin is higher than that of starch, resulting in a positive effect on the bending modulus, too.

Generally, based on the results reported in Table 2, the measured mechanical properties of the wood-based panels containing OSTL resin with DES-modified lignin were higher than the values required by the European Standard (EN-312). This shows the important effect of the DES modification of lignin in OSTL resin preparation. Furthermore, the positive influence of urea in the mechanical properties of the OSTL resin should not be ignored. The addition of urea as a cross-linker and co-reactant can also enhance the mechanical properties of the panels prepared by increasing the degree of crosslinking through the reaction of its –NH_2_ groups with some of the functional groups of both lignin and oxidized starch. This further enhances the cohesive bond strength of the adhesive. It must also be considered that increasing the level of oxidation of the starch does increase the cross-linking density of starch oxidized with lignin, as already shown in reference [21].

### 3.4. Water Absorption and Thickness Swelling

So far, several methods have been proposed to overcome the poor dimensional stability of wood panels [34,35]. One of the best methods proposed is the use of hydrophobic monomers/oligomers such as lignin in the structure of the resin. Table 2 shows that increasing the amount of unmodified/modified lignin from 10% to 30% in the OSTL resins progressively decreases both the thickness swelling and water absorption percentages of the panels. The sensitivity to water in the OSTL resin prepared is mainly due to the presence of the oxidized starch. The panels’ swelling decreases when adding lignin, as the residual -OHs of the starch can be consumed, and probably a further contribution to an increase in crosslinking can be obtained. As the hydrophobicity of lignin is somewhat similar to that of other phenolic oligomers, this is the main parameter inducing the reduction in both thickness swelling and water absorption.

Conversely, Table 2 also shows that at 30 wt% modification with DES-modified lignin, the prepared resin yields panels with the lowest water absorption (35%) and thickness swelling (13%), while the resin with 10% unmodified lignin had the highest water absorption (57%) and thickness swelling (33%). This can probably be ascribed to an increased number of reactive sites and a higher proportion of reactive groups such as phenolic hydroxyl groups in the lignin molecules induced by DES treatment. This may result in a higher reactivity of the lignin with oxidized starch. Moreover, by adding the DES-modified lignin to the OSTL resin, new chemical bonds can form between the reactants, hence reducing the proportion of the bridges sensitive to hydrolysis in the starch structure. Younesi-Kordkeili et al. (2024) reported that a DES treatment of lignin significantly decreased water absorption and thickness swelling of the panels bonded with PF and UF [28]. Finally, it can be noted that the addition of urea to OSTL as an active cross-linker significantly improves the dimensional stability of the particleboard panels, too.

As, however, ChCl-ZnCl_2_ DES is relatively expensive, in depth research work to decrease its inherent cost has been conducted [36,37,38]. A few different recycling procedures to minimize costs have been investigated, showing that after ten recycling steps, the regenerated DES conserves its initial effectiveness [36]. This work suggests that the recycling method presented may be a green, low-cost procedure for biomass pretreatment, rendering the use of DES economically possible for industrial applications.

## 4. Conclusions

In this work, a new bio-sourced adhesive system based on a ChCl–ZnCl_2_-based DES-modified lignin and oxidized starch is presented. The following conclusions can be drawn from the experimental results obtained:

- The poor dimensional stability and weak mechanical strength of the particleboard panels bonded with the lignin-oxidized starch resin could be improved by modifying lignin with DES in the formulation.
- FTIR analysis showed that the content of carboxyl groups at the 1200 cm^−1^ band increased after curing the OSTL resin. Even the content of carbonyl groups in the OSTL resin was lower than that in the OST resin.
- DSC analysis indicated that the curing temperature of the OSTL resin containing DES-modified lignin was lower than that with unmodified lignin.
- The panels containing the OSTL resin with DES-modified lignin presented higher dimensional stability and mechanical strength compared to those prepared with unmodified lignin.
- Higher dimensional stability and mechanical strength could be achieved by increasing the amount of DES-modified lignin in the OSTL wood adhesive from 10 to 30 wt%.

Finally, it must be considered that research on the use of DES-modified lignin is ongoing in several laboratories worldwide and that the work presented here presents just one of the possibilities for using DES-modified lignin for an industrial application. The future of a considerably significant application such as for wood panel adhesives and its future prospects is economically and technically dependent not only on the capability of DES recycling, but also on the search for and development of less expensive DESs.

## Figures and Tables

**Table 1:** The mechanical properties of the particleboard bonded with OSTL pristine and DES-modified lignin.

Resin	Bending Strength (MPa)	Bending Modulus (MPa)	IB Strength (MPa)
EN312	>11	>1800	≥0.40
OSTL + 10% Lignin	8.3 ± 0.01	1632 ± 70	0.35 ± 0.02
OSTL + 20% Lignin	7.2 ± 0.02	1520 ± 61	0.31 ± 0.03
OSTL + 30% Lignin	5.5 ± 0.03	1402 ± 42	0.27 ± 0.02
OSTL + 10% DES-modified Lignin	10 ± 0.05	1845 ± 33	0.39 ± 0.03
OSTL + 10% DES-modified Lignin	11.9 ± 0.03	2350 ± 51	0.41 ± 0.01
OSTL + 10% DES-modified Lignin	12.2 ± 0.04	2593 ± 50	0.44 ± 0.03

**Table 2:** Physical properties of the particleboard prepared.

Resin	Density	Water Absorption	Thickness Swelling
	(kg/m^3^)	(%)	(%)
OSTL + 10% lignin	750 ± 7	57 ± 2	33 ± 4
OSTL + 20% lignin	752 ± 4	53 ± 4	22 ± 2
OSTL + 30% lignin	751 ± 2	49 ± 3	16 ± 3
OSTL + 10% DES-modified lignin	749 ± 3	52 ± 6	28 ± 3
OSTL + 20% DES-modified lignin	750 + 5	45 ± 3	17 ± 2
OSTL + 30% DES-modified lignin	752 ± 4	35 + 2	13 ± 1

## Data Availability

The original contributions presented in this study are included in the article/Supplementary Material. Further inquiries can be directed to the corresponding authors.

## Supplementary Materials

The following supporting information can be downloaded at: https://www.mdpi.com/article/10.3390/polym17223023/s1, Table S1: Interpretation of MALDI ToF spectrometry peaks of DES-modified lignin with urea.
