# Supplementary material for: Wood Bio-Adhesives Made by Polymerizing Oxidized Starch with Deep Eutectic Solvent-Modified Lignin
Source: Polymers (Basel). 2025 Nov 14;17(22):3023. doi: 10.3390/polym17223023 (PMC12656508; doi:10.3390/polym17223023)
Supplement: Supplementary file 1 [file polymers-17-03023-s001.zip › polymers-3976807-supplementary.pdf]

SUPPLEMENTARY MATERIALS

Wood Bio-Adhesives by Polymerizing Oxidized Starch with Deep Eutectic Solvent-Modified Lignin

H.Younesi-Kordkheili, A.Pizzi

Table S1. Assignements of species from MALDI ToF spectra of the reaction of urea and DES-modified Lignin.

175 Da = with Na+	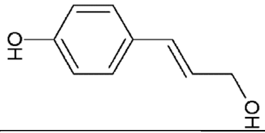
196 Da = without Na+	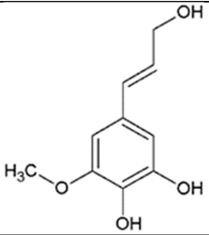
214 Da = one reacted urea, with Na+, deprotonated	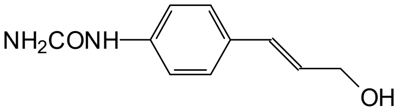
232 Da = with Na+	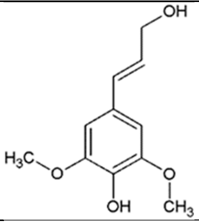
3<br><br>300 Da = with Na+	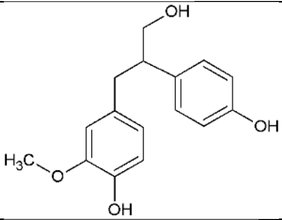
312 Da = with Na+, deprotonated	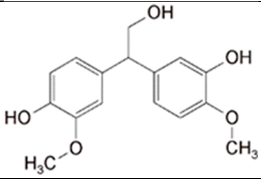
322 Da = no Na+, urea-linked Dimer, deprotonated	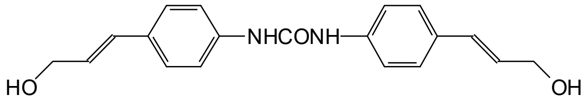
345 Da = urea-linked Dimer but with Na	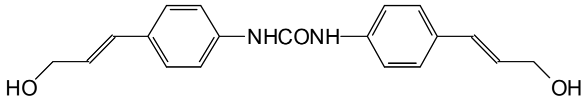

360 Da = no Na+	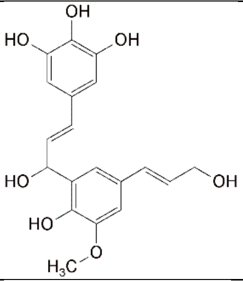
364 Da = with Na+, deprotonated	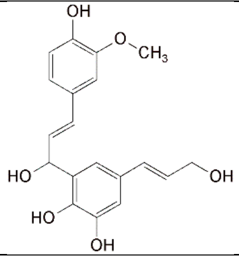
408 Da = deprotonated, with Na+, one urea reacted	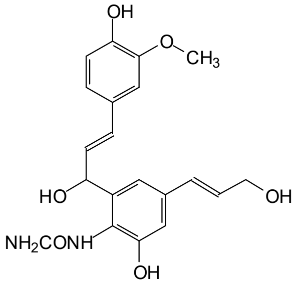
429 Da = with Na+	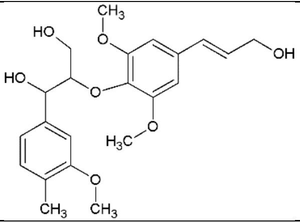
458 Da = no Na+	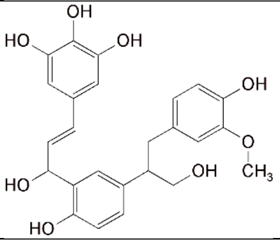
462 Da = one urea reacted, no Na+, deprotonated	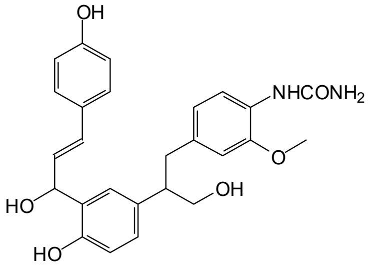
484 Da = one urea reacted , with Na+, protonated (same as above but with Na+)	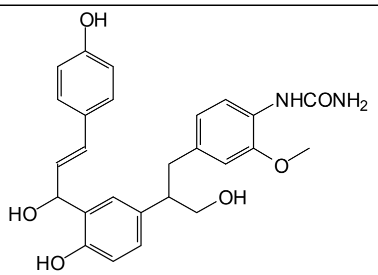

502 Da = 2 reacted ureas, no Na+, deprotonated	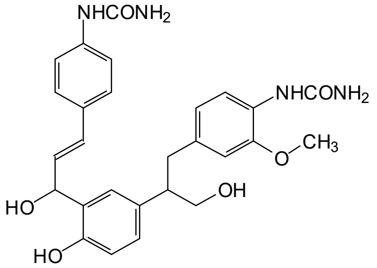
542 Da = 2 reacted ureas, with Na+, deprotonated	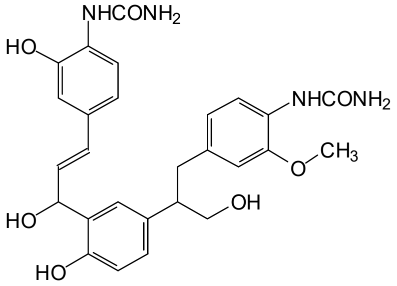
572 Da = 2 reacted ureas, no Na+, deprotonated	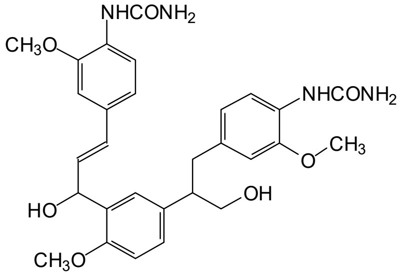
592 Da= 2 reacted ureas, deprotonated, with Na+	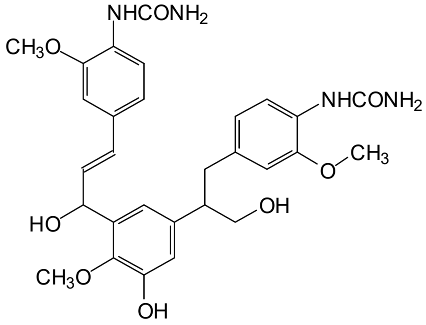
